# Evaluation of a parasite-density based pooled targeted amplicon deep sequencing (TADS) method for molecular surveillance of *Plasmodium falciparum* drug resistance genes in Haiti

**DOI:** 10.1371/journal.pone.0262616

**Published:** 2022-01-14

**Authors:** Swarnali Louha, Camelia Herman, Mansi Gupta, Dhruviben Patel, Julia Kelley, Je-Hoon M. OH, Janani Guru, Jean F. Lemoine, Michelle A. Chang, Udhayakumar Venkatachalam, Eric Rogier, Eldin Talundzic

**Affiliations:** 1 Oak Ridge Institute for Science and Education, Oak Ridge, TN, United States of America; 2 Centers for Disease Control and Prevention Foundation, Atlanta, GA, United States America; 3 Williams Consulting LLC, Atlanta, GA, United States America; 4 Division of Parasitic Diseases and Malaria, Malaria Branch, Center for Global Health, Centers for Disease Control and Prevention, Atlanta, GA, United States America; 5 The Wallace H. Coulter Department of Biomedical Engineering, Georgia Institute of Technology, Atlanta, GA, United States America; 6 Programme National de Contrôle de la Malaria, MSPP, Port-au-Prince, Haiti; National Institute of Research in Tribal Health, INDIA

## Abstract

Sequencing large numbers of individual samples is often needed for countrywide antimalarial drug resistance surveillance. Pooling DNA from several individual samples is an alternative cost and time saving approach for providing allele frequency (AF) estimates at a population level. Using 100 individual patient DNA samples of dried blood spots from a 2017 nationwide drug resistance surveillance study in Haiti, we compared codon coverage of drug resistance-conferring mutations in four *Plasmodium falciparum* genes (*crt*, *dhps*, *dhfr*, and *mdr1*), for the same deep sequenced samples run individually and pooled. Samples with similar real-time PCR cycle threshold (Ct) values (+/- 1.0 Ct value) were combined with ten samples per pool. The sequencing success for samples in pools were higher at a lower parasite density than the individual samples sequence method. The median codon coverage for drug resistance-associated mutations in all four genes were greater than 3-fold higher in the pooled samples than in individual samples. The overall codon coverage distribution for pooled samples was wider than the individual samples. The sample pools with < 40 parasites/μL blood showed more discordance in AF calls for *dhfr* and *mdr1* between the individual and pooled samples. This discordance in AF estimation may be due to low amounts of parasite DNA, which could lead to variable PCR amplification efficiencies. Grouping samples with an estimated ≥ 40 parasites/μL blood prior to pooling and deep sequencing yielded the expected population level AF. Pooling DNA samples based on estimates of > 40 parasites/μL prior to deep sequencing can be used for rapid genotyping of a large number of samples for these four genes and possibly other drug resistant markers in population-based studies. As Haiti is a low malaria transmission country with very few mixed infections and continued chloroquine sensitivity, the pooled sequencing approach can be used for routine national molecular surveillance of resistant parasites.

## Introduction

Hispaniola is the only island in the Caribbean with reported malaria cases, and 21,430 confirmed malaria cases were reported in Haiti in 2016 [1,2]. The primary vector transmitting malaria is *Anopheles albimanus* and approximately 98.5% of malaria in Haiti is *Plasmodium falciparum*, with *P*. *vivax* and *P*. *malariae* reported sporadically [3]. With Haiti’s national goal of interruption of local transmission of malaria by 2025, scale up of rapid diagnostic test use, investigation of drug resistance, and parasite elimination efforts have recently been accelerated [4,5].

Presently, chloroquine (CQ) with a single dose of primaquine is the primary treatment for uncomplicated *P*. *falciparum* malaria in the country [1], with the artemisinin combination therapy (ACT) artemether-lumefantrine [AL] replacing sulfadoxine-pyramethamine (SP) as the second line of treatment since April 2017 [6]. CQ remains efficacious against malaria in Haiti and offers many advantages as the country moves towards malaria elimination as it is inexpensive and generally well-tolerated [3,5]. Routine monitoring of the efficacy of the primary and secondary antimalarial treatments is a public health priority for Haiti as loss of these chemotherapeutics would pose additional challenges for malaria elimination [3,4].

Currently, a network of 11 sentinel health facilities in Haiti collect dried blood spot (DBS) samples from patients testing positive for *P*. *falciparum* malaria or patients enrolled in antimalarial drug-resistance surveillance. To date, no molecular markers for CQ resistance have been detected through this surveillance network [5]. A previous study has shown that along with the wild-type *crt* (CQ resistance) genotype, ~ 47% (257/548) of *P*. *falciparum* specimens had the *dhfr* S108N point mutation and one triple mutant was observed in this gene associated with pyrimethamine resistance [5]. Additionally, only a single isolate was found with a mutation A437G for the sulfadoxine resistance gene *dhps* [5].

Sanger sequencing has been utilized for drug resistance monitoring in Haiti to date [5], however, future work involves exploring the utility of using pooled Targeted Deep Amplicon Sequencing (TADS). Pooling of individual DNA samples prior to next generation sequencing could provide higher parasite densities and a more efficient strategy for estimation of allele frequencies (AFs) [7]. However, single nucleotide polymorphism (SNP) discovery and detection using pooled TADS can be accompanied by higher probabilities of sequencing errors, unless read coverage depth used to call SNPs is high [8]. In comparison, TADS of individual samples have lower probabilities of false AF calls [8], but is limited to a set number of samples (to obtain sufficient read coverage) and can be prohibitively expensive for large scale population-based studies. Thus, a specimen pooling strategy could be less efficient when coverage is low and individual sequencing is less efficient with higher expected read coverage [8].

To determine whether *P*. *falciparum* parasite density could have an effect on final AF estimation, 100 DNA samples from DBS obtained through a nationwide Haiti drug resistance surveillance study in 2017 were sequenced using two TADS methods: individually-sequenced DNA samples vs. pooled DNA sequencing. Specifically, the previously published individual patient sample TADS method [9] was modified to a pooling strategy based on parasite density of ten samples per pool. The sequencing data was used to analyze codon coverages for drug resistance-associated mutations in four *P*. *falciparum* genes, *crt*, *dhps*, *dhfr*, and *mdr1* (Table 1), and AF estimates based on parasite density were compared between the individual and pooled sequenced samples.

**Table 1:** Drug-resistance associated mutations in *P*. *falciparum* genes *crt*, *mdr1*, *dhps*, and *dhfr*.

Resistance to treatment	Gene	Most common mutations associated with drug resistance										
Chloroquine	*crt*	C72S	V73V	M74I	N75E	K76T	A220S	Q271E	N326S	C350S	I356T	R371I
Chloroquine, quinolone, mefloquine, amodiaquine, halofantrine, artemisinin combination therapy	*mdr1*	Y184F	S1034C	N1042D	D1246Y	N86Y						
Sulfadoxine-pyrimethamine	*dhps*	S436A/F/H	A437G	K540E	A581G	A613S/T						
Sulfadoxine-pyrimethamine	*dhfr*	N51I	C59R	S108N								

Talundzic et al. Antimicrob Agents Chemother. 2018. 62(4): e02474-17.

## Materials and methods

### Human subjects

Project was approved by Haitian IRB and not considered human subjects research by CDC Human Subjects Office (2015–155). Dried blood on filter paper was collected from March 2016- December 2017 from five health facilities in Haiti from symptomatic persons testing positive for *P*. *falciparum* malaria by rapid diagnostic test, CareStart Malaria HRP2 (Pf) (CareStart^TM^, Access Bio, USA). Patients of all ages were enrolled after consenting to test for molecular markers of malaria drug resistance. Whole blood was collected by finger prick on Whatman 903 Protein Saver cards (GE Healthcare). Each filter paper was air-dried overnight, stored in a desiccant containing bag at 4°C until shipment to CDC in Atlanta, GA. A 100 samples previously described [5] were chosen based on their Ct values for this method validation study. These samples had Ct values ranging from 21–40, and were selected since this Ct range had a wide distribution of parasites/μL (~ 0.2–71,000 parasites/μL).

### DNA extraction

Two 6mm punches from dried blood spots (DBS) were used to extract genomic DNA using the Qiagen DNA Mini extraction kit following the manufacturer’s instructions (QIAGEN, Valencia, CA). The DNA was eluted in 150μL of elution buffer aliquoted and stored at -20°C until use.

### Parasite density estimation using PET-PCR

Following DNA extraction, PET-PCR was performed as described previously on 100 samples in duplicate [10,11]. Cycle threshold (Ct) values above 40.0 was considered to be *P*. *falciparum* negative. The 3D7 *P*. *falciparum* culture strain was used as a positive control on PET-PCR assay plates, and in a ten- and two-fold dilution series (100,000 p/μL to 0.1 p/μL and 50 p/μL to 1.56 p/μL, respectively) to estimate parasitemia levels based on PET-PCR Ct values for each individual sample. Groups of ten samples with similar parasitemia values within 1.0 Ct values of each other were used to develop sample pools (10:1 ratio), and an average Ct value and mean estimated parasite density was calculated for each pool of ten samples (Table 2).

**Table 2:** Pooling of samples based on parasitemia levels.

Pool #	PET-PCR Ct [average]	Parasites per μL [estimate]
1	22.20	30,000
2	24.00	10,000
3	25.70	3000
4	27.90	600
5	29.80	150
6	31.80	40
7	34.10	10
8	36.00	3
9	37.80	1.0
10	40.0	0.4

Ten sets of 10:1 ratio sample pools were made based on similar Ct (estimated parasitemia) values. Ct values with corresponding parasitemia levels (parasites/μL) are shown.

### PCR enrichment of *crt*, *dhps*, *dhfr* and *mdr1*

PCRs were performed to amplify the four full-length *P*. *falciparum* genes for 100 individual DBS patient samples [9]. The *P*. *falciparum* 7G7 and HB3 culture strains were used as positive controls for comparison. The NEB High Fidelity PCR kit (New England BioLabs, USA) was used to amplify the genes according to the manufacturer’s instructions with a 50 μL master mix preparation using the 5× GC buffer.

### Individual patient and pooled patient targeted amplicon deep sequencing (TADS)

The full-length drug resistance genes (*crt*, *dhps*, *dhfr* and *mdr1)* for the 100 DNA samples were sequenced using the previously described Malaria Resistance Surveillance (MaRS) TADS protocol [9]. For individual patient level sequencing, unique sequence indices were added to the PCR amplicons, prior to pooling all the samples based on patient IDs, and sequenced on a single run. For the pooled level sequencing, samples with similar parasitemia values within 1.0 Ct values of each other (Table 2) were used to develop pools containing 10 individual DNA samples (10:1) prior to adding unique sequence indices, and then sequenced twice in two separate runs. Illumina-supported sequencing adaptors and unique sequence indices were added to the individual DNA or the pooled DNA lots using the Illumina Nextera XT kit (Illumina, USA). This step generates a unique sequence barcode identifier (ID) for each individual patient sample or pooled sample lot. The Nano MiSeq Reagent Kit V2 250 by 250 base pair (Illumina) was used for all three sequencing runs.

### Data analysis and visualization

For each of the four genes (*crt*, *dhps*, *dhfr* and *mdr1)*, a sample was considered as a ‘sequencing success’ if at least 50% of codons in the gene were covered by high-quality sequencing reads. Reads were categorized as ‘high-quality’ only when they had a Q20 > 99% (probability of 1 in 100 bases being called incorrect). High-quality reads were used by the next-generation sequencing analysis toolkit (NeST) (https://github.com/CDCgov/MaRS) to call non-synonymous single nucleotide polymorphisms (SNPs) in *crt*, *dhps*, *dhfr* and *mdr1*, using the same quality threshold cut-offs for the individual and pooled sequenced samples. All SNPs called by NeST were visually confirmed using the Geneious Prime software (www.geneious.com). Data visualization was performed using the python seaborn 0.10.1 package (https://seaborn.pydata.org).

### Individual vs. pooled sequence comparison

All the individual samples were sequenced using the same procedure used to process and sequence the pooled samples. Individual sequenced sample AFs were used to determine the expected AF of the 10:1 pooled samples as follows: individual AF per sample were added together and then divided by 10 to estimate the expected pooled AF. AF here refers to SNP AF, and not population-based AF. Importantly, all samples were mono infections as previously described by molecular barcoding [12] and only major AFs (>99%) in *dhfr (*N51I, C59R and S108N) and *mdr1* (Y184F, S1034C, N1042D, and D1246Y) were used for this analysis. The SNPs associated with anti-malarial drug resistance in *crt* and *dhps* were wild-type and AF calls for these genes were excluded from this analysis.

## Results

**Fig 1:**
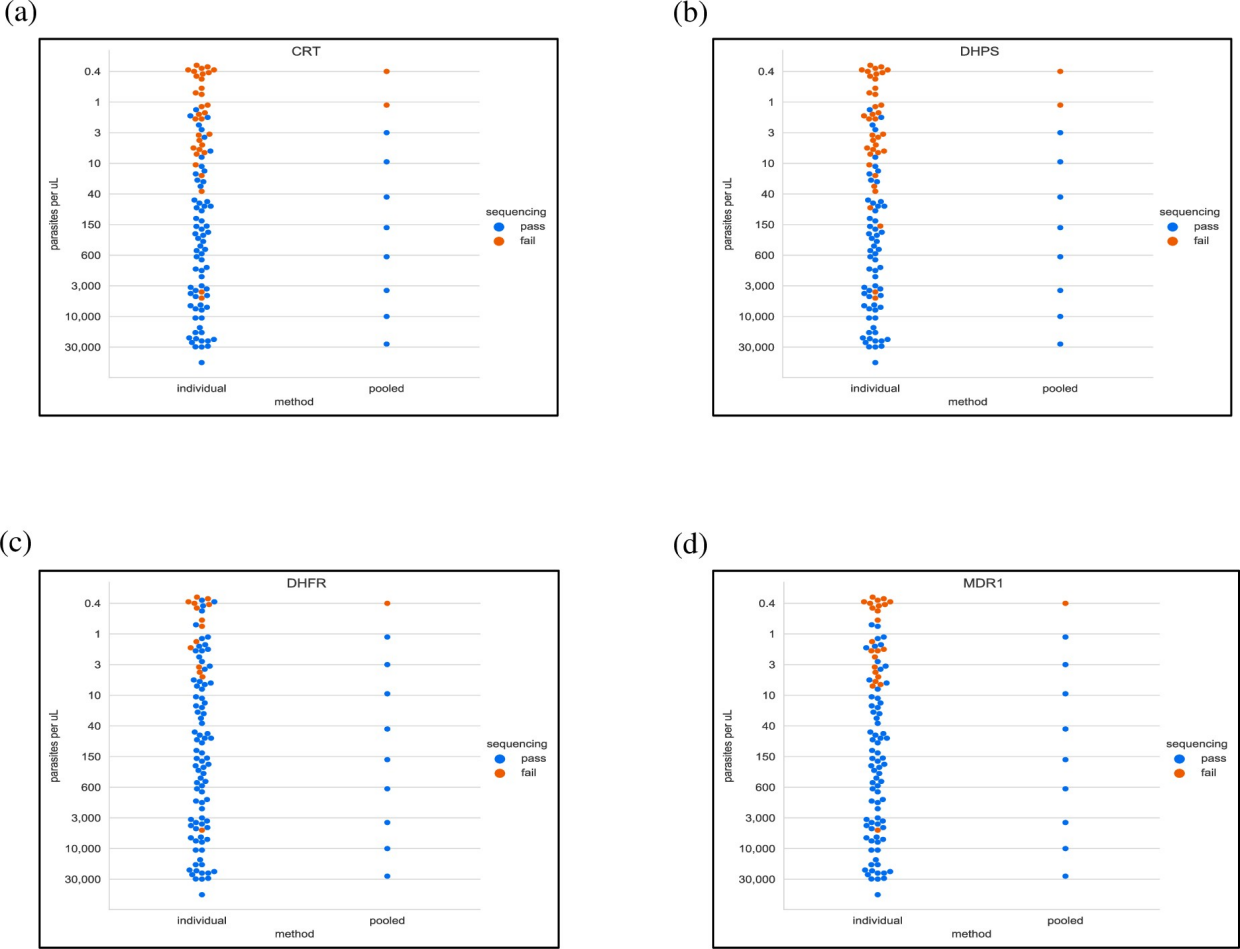
Sequencing outcome for four drug resistance genes between individual and pooled sequenced samples. Sequencing success (blue dot) or failure (orange dot) is determined by measuring the codon coverage of high-quality reads obtained. Sequencing method (individual vs pooled) is shown on the *x* axis and parasitemia levels (parasites/μL) are shown on the *y* axis. Individual sequenced samples = 100 total samples; same samples are pooled at 10:1 ratio = total of 10 sequenced sample pools.

The individual samples sequence method yielded high-quality reads for 68% (68/100) *crt* and 62% (62/100) *dhps* samples, whereas the pooled samples sequence method yielded high quality reads for 80% (80/100) of samples for both genes (Fig 1A and 1B). A higher percentage of samples yielded high quality reads for *dhfr* and *mdr1* by both the individual (dhfr:86% samples, mdr1:77% samples) and pooled (dhfr & mdr1 each: 90% samples) samples sequence methods (Fig 1C and 1D). Secondly, the pooled sequenced samples had a higher sequencing success at a lower parasite density (*crt* & *dhps*: > 1 parasite/μL; *dhfr* & *mdr1*: > 0.4 parasites/μL) than the individual samples sequence method (*crt* & *dhps*: > 40 parasite/μL; *dhfr* & *mdr1*: > 10 parasites/μL) (Fig 1).

**Fig 2:**
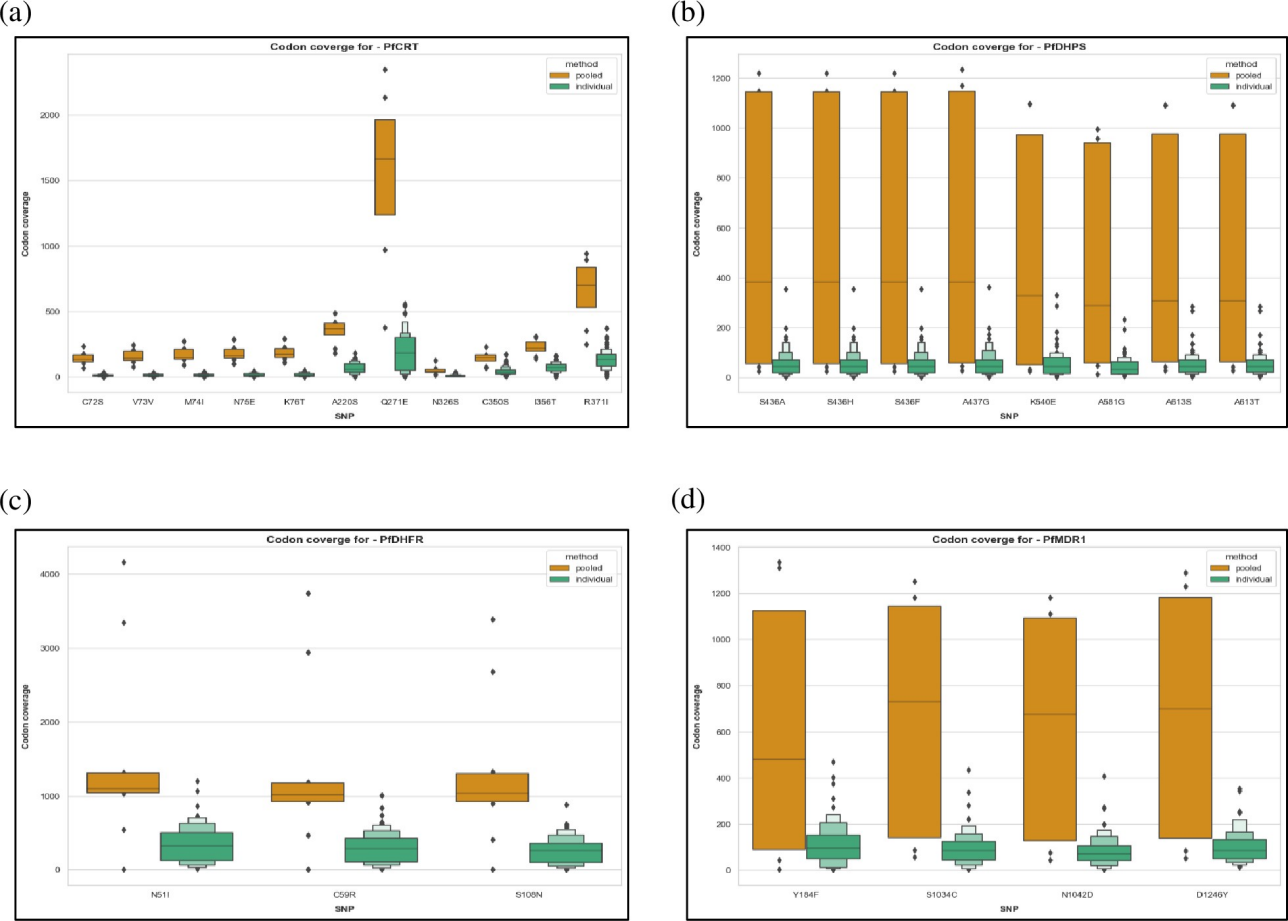
Comparison of codon coverage estimates for drug resistance-conferring SNPs between individual and pooled sequenced samples. SNP loci are shown on the *x* axis and codon coverage of read depth on the *y* axis. Orange: pooled sequenced samples; green: individual sequenced samples. Boxen plot distribution values: black centerline = median, which separates the upper 50^th^ percentile from the lower 50^th^ percentile of the dataset; each successive level outward from the median splits the remaining data further into two separate regions, denoting the 25^th^, 12.5^th^, and 6.25^th^ percentiles.

The median codon coverage for the *dhfr* anti-malarial drug resistance associated mutations N51I, C59R and S108N had a greater than 3-fold increase from individual to pooled samples (Fig 2C and S1 File). In comparison, the *dhps* mutations (S436A, S436H, S436F, A437G, K540E, A581G, A613S, and A613T) and the majority of *mdr1* mutations (S1034C, N1042D, and D1246Y) had a greater than 7-fold increase in median codon coverage (Fig 2B and 2D and S1 File). The highest increase in median codon coverage were in the *crt* mutations C72S, V37V, M74I, N75E, and K76T, which had a greater than 12-fold increase from individual to pooled sequenced samples (Fig 2A and S1 File). The overall codon coverage distribution for the pooled sequenced samples based on estimated parasite densities was wider than the individual samples (Fig 2). Here, the number of pools considered for calculating the codon coverage distribution of the pooled sequenced samples for each gene depended on the codon coverage of drug-resistance associated mutations obtained in each of the pools. For example, for all four genes, pools nine and ten, which had the lowest parasite densities and thus negligible codon coverage were omitted from the analysis (S2 File).

**Fig 3:**
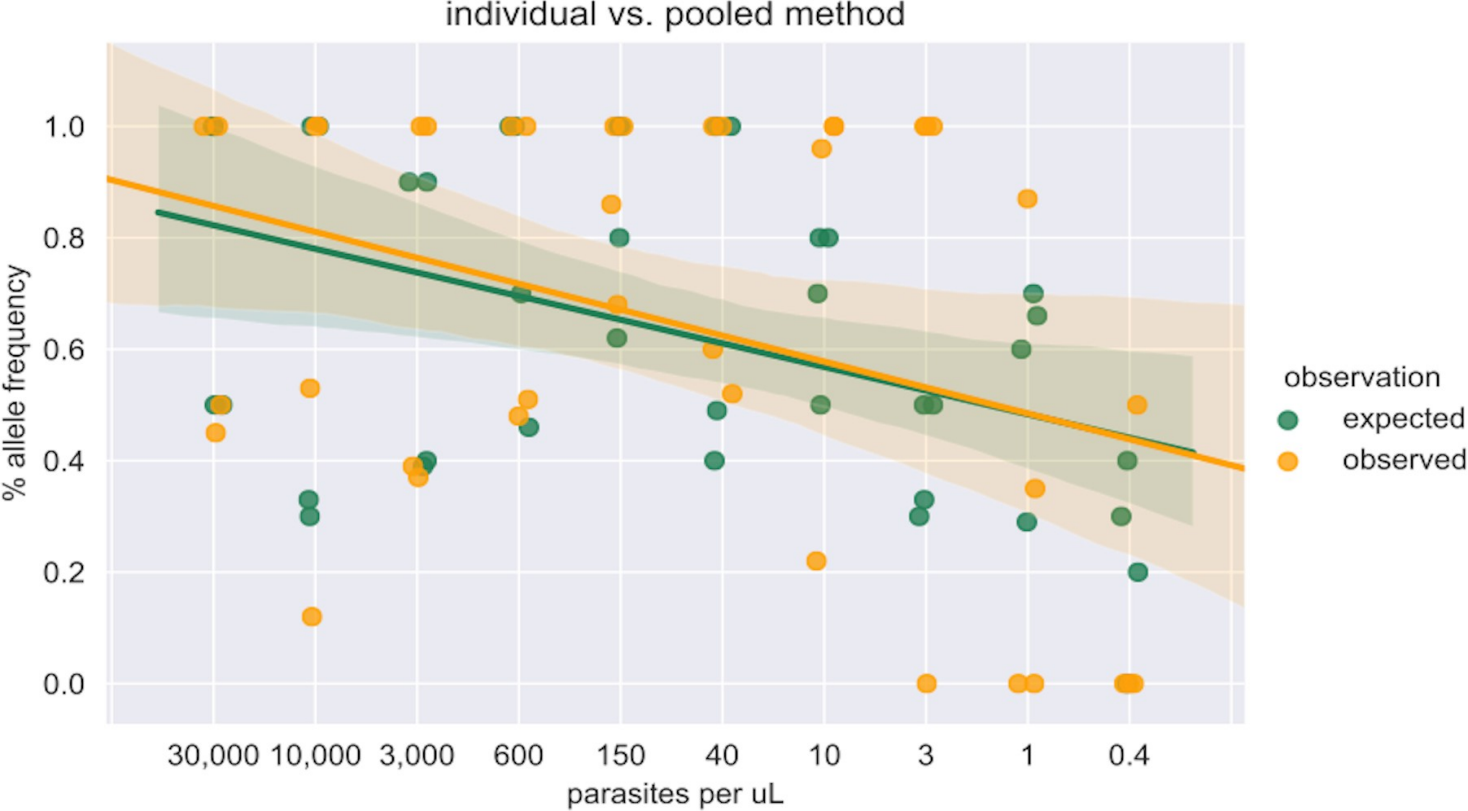
Allele frequency comparison between individual and pooled sequenced samples for *dhfr* and *mdr1*. Parasitemia levels (parasites/μL) are shown on *x* axis. Percent allele frequencies are shown on the *y* axis. Green dots: individual sequenced samples allele frequency (expected allele frequency); orange dots: pooled sequenced samples allele frequency (observed allele frequency). Green/orange line = best fit, green/orange highlighted region = confidence bounds.

The mean AFs of the wild-type and mutant alleles for each of the four genes in the individual and pooled samples are provided in Table 3, where the AFs for the pooled samples are based on weighted averages of parasitemia levels in each of the pools (S3 File). Comparison of AF estimates for *dhfr* and *mdr1* between the individual and pooled sequenced samples as a measure of parasitemia indicates that at lower parasite densities (< 40 parasites/μL), the AF calls of pooled sequenced samples are not accurately estimated when compared to the AF calls of individual sequenced samples (i.e. expected AF calls) (Fig 3).

**Table 3:** Mean allele frequencies of wild type- and mutant allele-associated drug resistance SNPs in individual and pooled samples.

	Individual DBS		Pooled DBS		Difference in percent allele frequency between Individual and Pooled DBS	*p*-value (α = 0.05)
Gene and wild type allele	Sample coverage (n)	Mean allele frequency (%)	Sample coverage (n)	Mean allele frequency (%)		
***crt*:**						
C72	87	98.85	80	100.00	1.15	0.38
K76	88	98.86	80	100.00	1.14	0.38
A220	86	98.84	80	100.00	1.16	0.38
Wild type ^a^	varies, from 86–90	100.00	80	100.00	0	-
***dhps*:**						
S436	82	98.78	80	100.00	1.22	0.38
S436	82	100.00	80	100.00	0	-
S436	82	100.00	80	100.00	0	-
A437	82	95.12	80	100.00	4.88	0.27
Wild type ^b^	varies, from 78–82	100.00	80	100.00	0	-
***dhfr*:**						
N51	94	98.71	90	100.00	1.29	0.36
C59	94	100.00	90	100.00	0	-
S108 ^c^	94	46.59	90	65.56	18.97	0.49
***mdr1*:**						
Y184 ^c^	86	6.98	90	0	6.98	0.06
S1034	87	98.85	90	100.00	1.15	0.37
N1042	86	97.67	90	100.00	2.33	0.32
D1246	84	98.81	90	100.00	1.19	0.37

The allele frequencies in pooled samples are weighted averages of mean parasitemia levels in each pool. The *p*-values of the difference in mean allele frequencies between individual and pooled samples have been tested at α = 0.05.

^a^ wild type alleles V37, M74, N75, Q271, N326, C350, I356, and R371.

^b^ wild type alleles K540, A581, and A613.

^c^ greater than 50% mutations were only found for S108N (53%) and Y184F (97%).

DBS: dried blood spot.

n changes as Coverage distribution is not uniform across the gene.

## Discussion

Pooling of malaria samples prior to TADS has been shown to be a cost-effective and time saving approach for estimating drug resistance associated mutations in *P*. *falciparum*, as it reduces the number of PCR reactions and library preparations [7,13–15]. One pooling strategy is to combine DBS from individual samples for DNA extraction prior to nested PCRs and TADS [14], while another strategy involves combining genomic DNA (gDNA) in varying pool ratios (i.e. 35 to 273) prior to TADS [15]. Although large sample sizes in pools can theoretically help in determining robust AFs due to a decrease in overall variance of AF estimates [15], both these strategies [13,14] can result in biased AFs due to varied parasite densities within individual samples without properly achieving equimolar concentrations of individual DNA in the pools. This is especially the case with alleles present at low frequency in pools that can lead to false positive variants [16].

Here, in an effort to minimize some of these potential biases due to parasite densities and increase the power of genetic analysis, we evaluate a new pooling strategy by using estimated parasite densities to guide the pooling of DBS-derived gDNA in 10:1 ratio prior to TADS. Pooling samples based on similar parasite densities allows direct calculation of allele frequencies in the parasite population rather than identifying the prevalence of individuals bearing parasites with mutated alleles. [14]. The ten to one ratio was chosen to limit further biases introduced to AF due to library preparation chemistries [17] and reduction in sequencing coverage, with the latter having the most influence on accurate AF estimation [18]. By grouping ten samples per pool, the total experiment remains cost efficient (10 vs 100 reactions) while providing more flexibility in overall study design (e.g., such as further grouping by treatment arm or regional site) [19]. Our strategy resulted in an overall increase in codon coverage of SNPs conferring antimalarial drug resistance consistently in pooled samples when compared to individually sequenced samples (Fig 2). This increase in coverage together with parasitemia-based pooling helped achieve reliable AF estimation in pools (Table 3) across different full-length drug resistance associated parasite genes, thus allowing the identification of novel SNPs in a population [9]. The minor difference in mean AFs observed between the individual and pooled sequenced samples in our study are statistically not significant (Table 3), but nevertheless also show the limitation of the pooled sequencing approach in detecting rare variants. This limitation can be considered an acceptable trade-off in light of the perceived relative advantages of pooling.

This study also provides evidence that AF estimation from pools of DNA can be under- or over-represented at low parasite densities (< ~40 parasites/μL, Fig 3), given that such estimates of parasitemia are based on PET-PCR Ct values such as those obtained in our study. This is because although pooled samples may show sequencing success at low parasite densities, multiple individual samples within these pools are in fact sequencing failures (Fig 1), and do not contribute towards AF estimation in the pools. Hence, below certain thresholds of parasite densities (< ~40 parasites/ μL for *crt* and *dhp*s, and < ~10 parasites/μL for *dhfr* and *mdr1*), AFs of sample pools can represent AFs of only a few individual samples which were successfully sequenced in those pools. The difference in parasite density thresholds in these four genes can be attributed to non-uniform sequencing coverage, which is influenced by factors such as GC content, read length and preferential amplification of certain DNA fragments [9].

While the added step of having to use a real-time PCR assay to estimate parasitemia values in samples may seem like a limitation of this strategy, it can serve as an important quality control step to assess DNA quantity and confirm or identify accurately the *Plasmodium* spp. in samples [10,20]. Using a reliable and accurate DNA quantification method prior to sequencing will also improve overall sequencing capacity and further reduce costs by avoiding either under or over usage of NGS capacity [21]. Loading of unbalanced indexed DNA libraries, commonly the case with pooling based strategies, may result in unnecessary data loss and further SNP estimation errors [21]. Thus, the added step of implementing a *Plasmodium-*specific DNA quantification step prior to pooling is an important part of an effective quality control system for NGS based surveillance tests [22].

One of the major limitations of most pooling strategies, including the work described here, is the inability to accurately identify reads that belong to individual samples in a pool or genotype select polymorphisms such as copy number variations of resistance associated genes like *mdr1* [12]. However, using first a pooling strategy to identify possible SNP signals of interest, especially novel SNPs that might be associated with drug resistance, followed by individual sample sequencing can be a good approach for confirming drug resistance in particular samples while keeping overall costs efficient. In this regard, limiting the number of samples to ten per pool can be considered as a good strategy, as individual sample sequencing for confirming drug resistant SNPs in larger pools can lead to increased costs.

In order for population-level data collected over different periods of time to be useful for meta-analysis, ideally the same laboratory pipeline (e.g., pooling strategy, amplification, library preparation and sequencing chemistries) and analysis should be used. This is especially important in malaria pre-elimination settings or routine therapeutic efficacy studies being conducted on a regular basis across different geographical regions or the same geographic region over time. Thus, the trade-off between cost savings and quality of data–especially reproducible data over time and space–should be considered when planning an NGS-based pooling strategy for molecular surveillance of drug resistance genes. An important finding of this study was the use of blood dried on filter paper (DBS) as an appropriate sample type for DNA pooling and subsequent TADS. As cold-chain requirements are not needed and blood collection can be done by finger stick, preparation and transport of DBS are much more logistically feasible in resource-limited settings [23,24]. DBS are increasingly being used as the sample type for surveys and studies of large sample sizes due to these practical advantages [25].

Recent reports from our laboratory and others [2,5,26,27] have demonstrated that the prevalence of chloroquine-resistant *crt* mutations has remained very low in Haiti. We chose 100 samples from our previously reported study [5] for the current NGS methods development and 99 of them were found to have no mutations in the *crt* gene. Only one sample was found to have chloroquine-resistant mutations at codons C72S, K76T, and A220S. We also found two samples with mutations at codons N51I in the *dhfr* gene, and 52 samples (~ 55% of all successfully sequenced samples) with the *dhfr* S108N mutations, which is associated with low level of resistance to pyrimethamine. For *mdr1*, 92% of successfully sequenced samples were found to have a single Y184F mutation and were wild type at all other interrogated loci. These observations are also in agreement with previous studies in Haiti [5,26–28]. Although a single S108N mutation is capable of conferring low-level resistance to pyrimethamine, presence of at least three mutations (in N51I, C59R, and S108N) can lead to high-level resistance.

Since SNP-based methods for detecting genetic variations have limited discriminatory power for identifying polyclonal infections [29–31], our pooled sequencing approach has limitations in calling appropriate SNPs in mixed infection samples. However, occurrences of mixed infections are rare in Haiti [32,33]. Specifically, for the molecular resistance surveillance program in Haiti, this study provides a clear proof-of-concept to move forward with a DNA pooling strategy for identification of codon mutations associated with antimalarial drug resistance [5]. Through this nationwide surveillance network, approximately 850 DBS are collected on an annual basis [6]. The ability to pool extracted DNA specimens at a ten to one ratio will greatly reduce the cost and time associated with gaining molecular estimates of *P*. *falciparum* haplotypes from large samples in Haiti.

## Supporting information

S1 File Median codon coverages of anti-malarial drug resistance genes in the individual and pooled samples. (XLSX)

S2 File Codon coverage of anti-malarial resistance genes obtained by both the individual and pooled sequencing methods in each of the 10 pools of samples. (DOCX)

S3 File Weighted average of parasitemia levels in each of the10 pools of samples. (XLSX)
