# Correction: Evaluation of a parasite-density based pooled targeted amplicon deep sequencing (TADS) method for molecular surveillance of Plasmodium falciparum drug resistance genes in Haiti

**DOI:** 10.1371/journal.pone.0315044

**Published:** 2024-12-02

**Authors:** Swarnali Louha, Camelia Herman, Mansi Gupta, Dhruviben Patel, Julia Kelley, Je-Hoon M. OH, Janani Guru, Jean F. Lemoine, Michelle A. Chang, Udhayakumar Venkatachalam, Eric Rogier, Eldin Talundzic

There is an error in affiliation #3 for all the authors. The correct affiliation #3 is: Malaria Branch, Division of Parasitic Diseases and Malaria, Center for Global Health, Centers for Disease Control and Prevention, Atlanta, GA, USA. The publisher apologizes for the error.

An additional affiliation is missing for the first author. Swarnali Louha is also affiliated with Malaria Branch, Division of Parasitic Diseases and Malaria, Center for Global Health, Centers for Disease Control and Prevention, Atlanta, GA, USA.

An additional affiliation is missing for the second author. Camelia Herman is also affiliated with Malaria Branch, Division of Parasitic Diseases and Malaria, Center for Global Health, Centers for Disease Control and Prevention, Atlanta, GA, USA.

An additional affiliation is missing for the third author. Mansi Gupta is also affiliated with Malaria Branch, Division of Parasitic Diseases and Malaria, Center for Global Health, Centers for Disease Control and Prevention, Atlanta, GA, USA.

There is an error in affiliation #2 for the fourth author, Dhruviben Patel. The correct affiliations #2 is: Williams Consulting LLC, Baltimore, MD, USA.

The affiliation for the fifth author is incorrect. Julia Kelley is not affiliated with #1 only with #3: Malaria Branch, Division of Parasitic Diseases and Malaria, Center for Global Health, Centers for Disease Control and Prevention, Atlanta, GA, USA.

An additional affiliation is missing for the sixth author. Je-Hoon M. OH is also affiliated with Malaria Branch, Division of Parasitic Diseases and Malaria, Center for Global Health, Centers for Disease Control and Prevention, Atlanta, GA, USA.

The following information is missing from the Competing Interests statement: The authors declare the following competing interests: Dhruviben Patel is a paid employee of Williams Consulting LLC, Baltimore, MD, USA. There are no patents, products in development or marketed products associated with this research to declare. This does not alter our adherence to PLOS ONE policies on sharing data and materials.
