# Supplementary material for: Evaluation of a parasite-density based pooled targeted amplicon deep sequencing (TADS) method for molecular surveillance of Plasmodium falciparum drug resistance genes in Haiti
Source: PLoS One. 2022 Jan 14;17(1):e0262616. doi: 10.1371/journal.pone.0262616 (PMC8759662; doi:10.1371/journal.pone.0262616)

Codon coverage of anti-malarial resistance genes obtained by both methods (individual and pooled sequencing) in each of the 10 pools of samples

*crt*:

*dhps*:
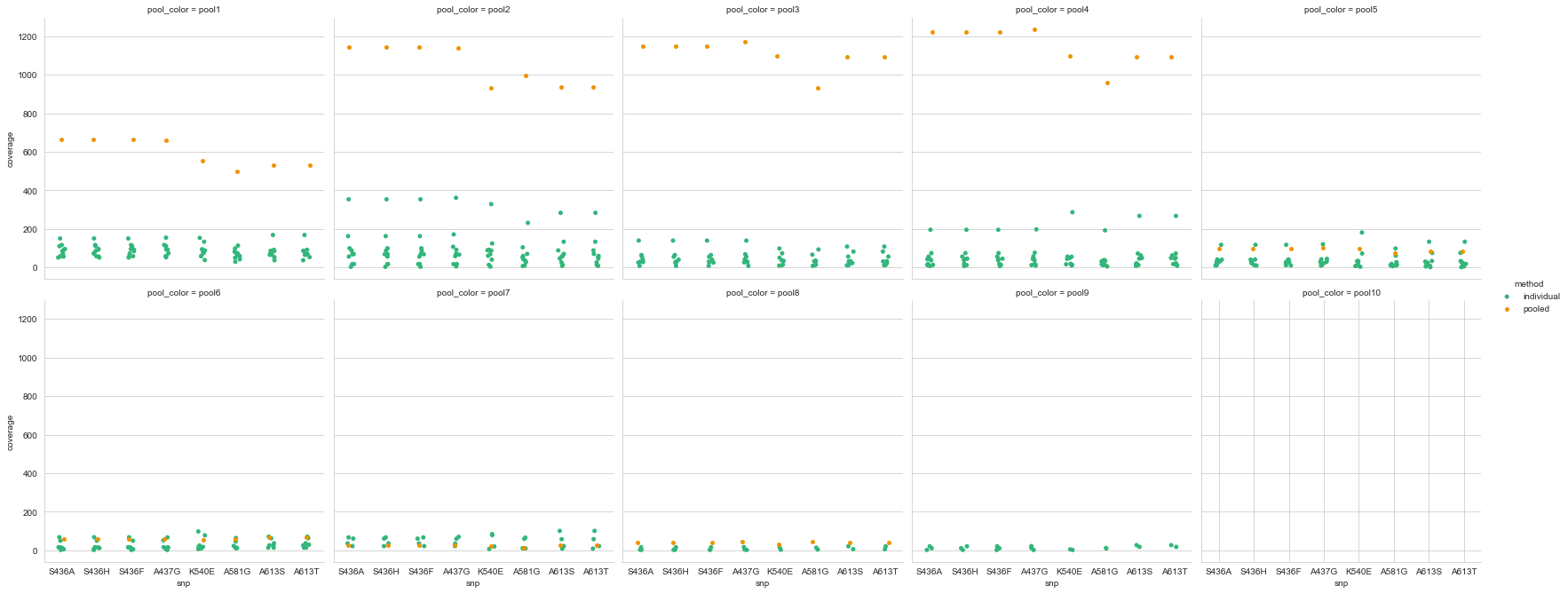


*dhfr*:


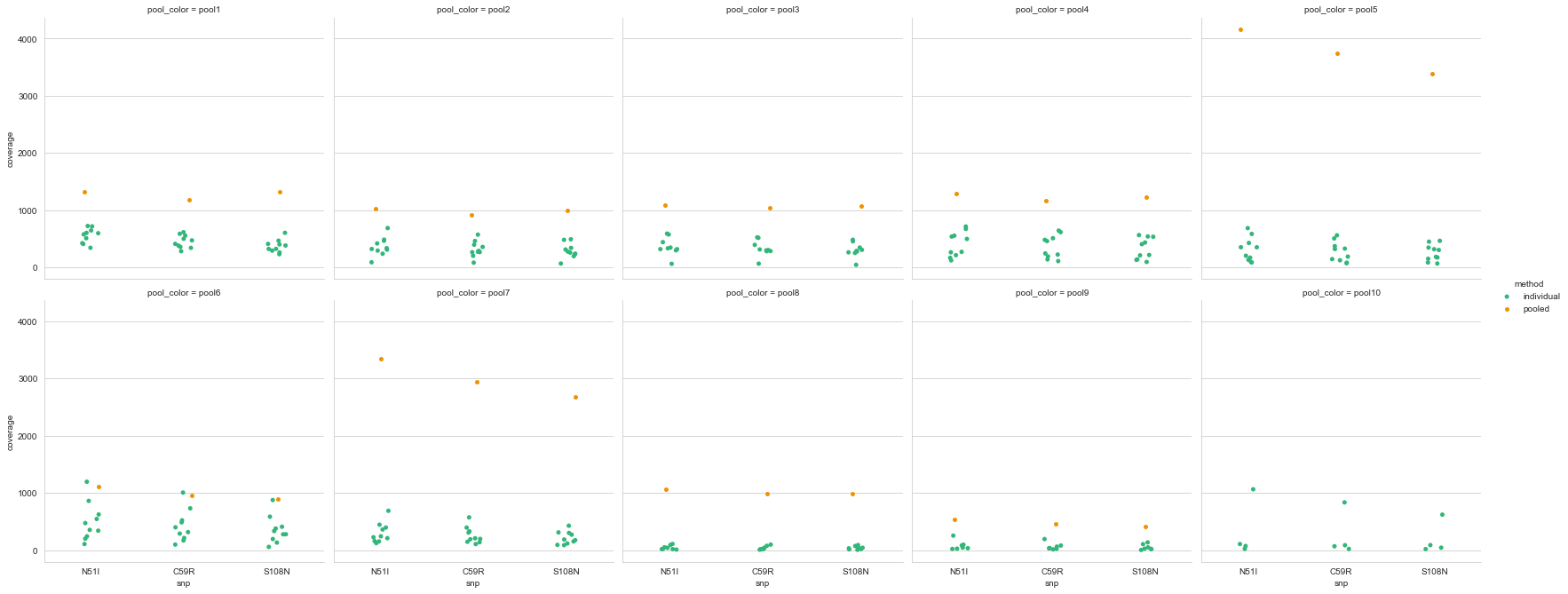


*mdr1*:


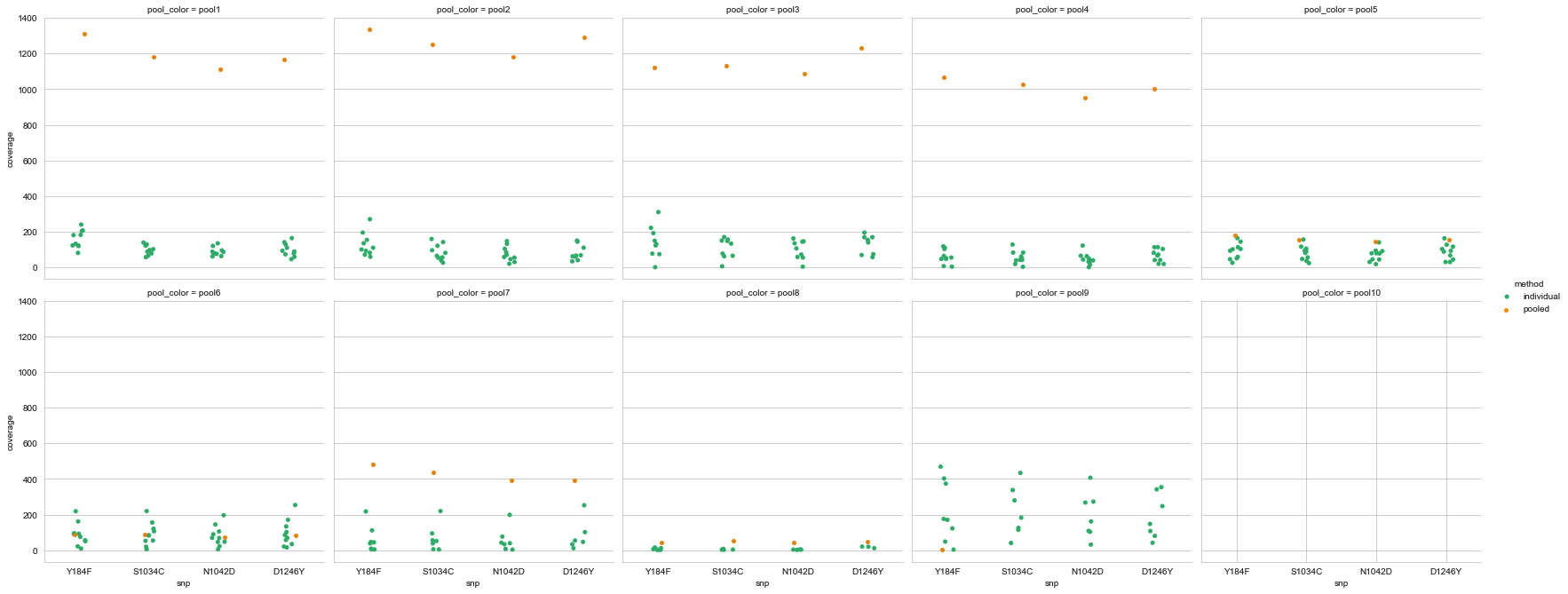

Supplement: S2 File — (DOCX) [file pone.0262616.s002.docx]
